# Modulations of the Chicken Cecal Microbiome and Metagenome in Response to Anticoccidial and Growth Promoter Treatment

**DOI:** 10.1371/journal.pone.0027949

**Published:** 2011-11-16

**Authors:** Jessica L. Danzeisen, Hyeun Bum Kim, Richard E. Isaacson, Zheng Jin Tu, Timothy J. Johnson

**Affiliations:** 1 Department of Veterinary and Biomedical Sciences, College of Veterinary Medicine, University of Minnesota, St. Paul, Minnesota, United States of America; 2 Minnesota Supercomputing Institute, University of Minnesota, St. Paul, Minnesota, United States of America; Hospital for Sick Children, Canada

## Abstract

With increasing pressures to reduce or eliminate the use of antimicrobials for growth promotion purposes in production animals, there is a growing need to better understand the effects elicited by these agents in order to identify alternative approaches that might be used to maintain animal health. Antibiotic usage at subtherapeutic levels is postulated to confer a number of modulations in the microbes within the gut that ultimately result in growth promotion and reduced occurrence of disease. This study examined the effects of the coccidiostat monensin and the growth promoters virginiamycin and tylosin on the broiler chicken cecal microbiome and metagenome. Using a longitudinal design, cecal contents of commercial chickens were extracted and examined using 16S rRNA and total DNA shotgun metagenomic pyrosequencing. A number of genus-level enrichments and depletions were observed in response to monensin alone, or monensin in combination with virginiamycin or tylosin. Of note, monensin effects included depletions of *Roseburia*, *Lactobacillus* and *Enterococcus*, and enrichments in *Coprococcus* and *Anaerofilum*. The most notable effect observed in the monensin/virginiamycin and monensin/tylosin treatments, but not in the monensin-alone treatments, was enrichments in *Escherichia coli*. Analysis of the metagenomic dataset identified enrichments in transport system genes, type I fimbrial genes, and type IV conjugative secretion system genes. No significant differences were observed with regard to antimicrobial resistance gene counts. Overall, this study provides a more comprehensive glimpse of the chicken cecum microbial community, the modulations of this community in response to growth promoters, and targets for future efforts to mimic these effects using alternative approaches.

## Introduction

For more than 50 years, antibiotic growth promoters (AGPs) have been used in agricultural animal production in the United States and other countries as a means to increase production through maintained animal health and improved feed efficiency. The ionophore monensin has been used by the broiler industries in the United States for over forty years to control coccidiosis in poultry [1]. Monensin has broad anticoccidial activity [2] and a mode of action targeted at the *Eimeria* parasite. In the United States broiler chicken and turkey industries, AGPs and monensin are commonly combined in feed at low levels. Despite the successes of such use in poultry, the underlying mechanisms responsible for these effects are not completely understood. It is assumed that modulation of the gut flora by constant low level presence of an antibiotic plays a role in the benefits conferred to the host [3].

The benefits of AGP use in production animals are often argued to be outweighed by their negative effects. For example, the use of AGPs has been associated with the emergence of pathogens resistant to fluoroquinolones, vancomycin, and third- and fourth-generation cephalosporins, among others [4], which has already led to a ban on AGP use in feed in the European Union [5]. Until recently, there has been little regulatory activity regarding AGPs in the United States; however, in 2005 the U.S. Food and Drug Administration banned the use of enrofloxacin in poultry due to an increase in fluoroquinolone-resistant *Campylobacter*, a trend that paralleled the increased use of the drug in the poultry industry [6]. Both political and consumer pressures are prompting a reduction in the use of AGPs in production animals, necessitating the identification of alternative approaches that will exhibit similar benefits to animals. Tylosin and virginiamycin are two antibiotics of interest because both are used in the U.S. poultry industry and have analogs in use (erythromycin and quinupristin-dalfopristin, respectively) for therapy against human pathogens. Erythromycin resistance in *Campylobacter jejuni* has been reported as high as 56.1% in broilers treated with subjected to subtherapeutic tylosin administration [7]. In addition, Kieke *et al*. reported 56% resistance in *Enterococci faecium* isolated from chicken and an association between poultry consumption and inducible quinupristin-dalfopristin resistance [8]. Because of these findings, efforts are now underway in the U.S. by many poultry producers to phase out antibiotics with human analogs in production animals, underscoring the need to better understand their impacts on gut flora.

A number of previous studies on poultry bacterial populations have relied on cultivation and enumeration of bacterial species [9]; more recently, PCR-based culture-independent methods have been employed in an effort to overcome the limitations and biases associated with culture-based techniques [10]. The most commonly used molecular methods rely on amplification of the 16S rRNA, such as denaturing gradient gel electrophoresis (DGGE) of the PCR-amplified 16S rRNA genes [11], [12], use of species-specific primers [13], or sequencing of randomly selected 16S rRNA clones [14]. Amplification of one or more hypervariable regions of the 16S rRNA region followed by parallel tag pyrosequencing is now commonly employed to analyze many different bacterial populations [15], [16]. In this study, we used pyrosequencing of the V3 hypervariable region and shotgun metagenomic sequencing to analyze the effects of subtherapeutic levels of two antimicrobials, virginiamycin and tylosin, and the anticoccidial monensin, on bacterial populations in the chicken cecum.

## Materials and Methods

### Sample Collection

All animal experiments were performed in accordance with the Institutional Animal Care and Use Committee at the University of Minnesota under protocol number 0807A39862. Two trials were performed using commercial day-of-hatch Ross x Ross chickens (n = 160) randomly separated into 4 groups of 40 birds. The groups were housed in separate pens in the same building in the Research Animal Facility at the University of Minnesota. The four groups were fed the same control diet without antibiotics until seven days of age, when three groups were switched to a diet containing subtherapeutic levels of monensin sodium (110 g/ton), or monensin sodium (110 g/ton) with virginiamycin (15 g/ton) or tylosin phosphate (20 g/ton), in accordance with FDA guidelines (http://www.fda.gov/); the fourth group remained on the control diet. At day 0 pre-treatment, and days 7, 14, and 35 post-diet alteration, 10 chickens were randomly selected from each group and humanely euthanized. Cecal contents were aseptically collected from each bird and immediately stored at −80°C and promptly processed.

### DNA Extraction

Cecal samples from the chickens were pooled together according to group and time point. DNA was extracted from pooled samples using a bead-beating procedure. Briefly, 0.25 g of pooled cecal content were suspended in 1 ml lysis buffer (500 mM NaCl, 50 mM Tris-Cl, pH 8.0, 50 mM EDTA, 4 % SDS) with glass beads, including 0.3 g of 0.1 mm size and 0.1 g of 0.5 mm size (Biospec Products, Bartlesville, OK), and homogenized on a bead-beater for 3 min at full speed. The samples were then heated at 70°C for 15 min, followed by centrifugation to separate the DNA from the bacterial cellular debris. This process was repeated with a second 300 ul aliquot of lysis buffer. The samples were then subjected to 10 M v/v ammonium acetate precipitation, followed by isopropanol precipitation and a 70% ethanol wash and resuspended in 100 ul 1× Tris-EDTA (Fisher Scientific, Fair Lawn, NJ). The samples were treated with DNase-free RNase (Roche, Basel, Switzerland) for 15 minutes at 37°C, and then processed through the QIAmp® DNA Stool Mini Kit (Qiagen, Valencia, CA) according to manufacturer's directions with some modifications. Samples were measured on a Nanodrop ND-1000 spectrophotometer (Thermo Scientific) to assess DNA quantity.

### 16S rRNA Amplification and 454 Sequencing

The V3 hypervariable region of the 16S rRNA gene was amplified in a 50 µl reaction containing 1× PCR buffer (containing 1.8 mM MgCl_2_), 0.2 mM each dNTP (Promega, Madison, WI), 0.4 µM each primer (Integrated DNA Technologies, Coralville, IA), 2.5 U FastStart High Fidelity Taq Polymerase (Roche), and 50 ng DNA template. The primers used were 5′-CCTACGGGAGGCAGCAG-3′ with adapter A (forward primer) and 5′-ATTACCGCGGCTGCTGG-3′ with adapter B (reverse primer), and sample-specific sequence barcodes designed by Roche (Technical Bulletin 013-2009) [17], [18]. The PCR conditions used were 95°C for 2 min; 20 cycles of 95°C for 30 sec, 60°C for 30 sec and 72°C for 30 sec; followed by 72°C for 7 min. Two amplification reactions were run for each sample and pooled together. The PCR product (approximately 230 bp) was excised from a 1.5% agarose gel stained with ethidium bromide and purification was performed using the QIAquick Gel Extraction Kit (Qiagen). DNA quality and concentration were assessed on a Bioanalyzer 2100 (Agilent, Palo Alto, CA) using a DNA 1000 lab chip. Barcoded samples were combined equal concentrations of 5 ng/µl and divided into 2 runs; pyrosequencing was carried out by the BioMedical Genomics Center at the University of Minnesota using GS FLX technology (Roche).

### Metagenomic Sequencing

Total DNA from pooled samples from the day 14 and day 35 post-treatment timepoints were subjected to shotgun metagenomic sequencing using GS-FLX sequencing with Titanium chemistry. Eight pooled samples (D14C, D14M, D14V, D14T, D35C, D35M, D35V, and D35T) were barcoded and sequenced on one full plate. The amplicon and metagenome reads used in this paper are publicly available from the SEED platform (http://metagenomics.anl.gov/).

### Data Analysis

Following sequencing, all barcodes were sorted, removed, and reads were quality assessed. To minimize effects of random sequencing errors, we eliminated 1) sequences that did not appropriately match the PCR primer and the barcode at the beginning of a read, 2) sequence reads with <50 bases after the proximal PCR primer if they terminated before reaching the distal primer, 3) sequences that contained more than one undetermined nucleotide (N), and 4) sequences with a average *phred* quality score of ≤27. Both the proximal and distal primers were trimmed from high-quality reads before database searches and similarity calculations. Then, the 16S rRNA sequences were quality screened and trimmed to identical beginning and end nucleotides extending from the end of the V3 universal primers. The RDP Database was used to assign reads to taxonomic groups with a bootstrap cut-off of 80% and perform statistical comparisons between groups [19]. The Mothur package [20] was used in operational taxonomic unit (OTU)-based analysis including rarefaction curves, dendrogram, Venn diagrams, and heat maps with an OTU definition at a similarity cutoff of 95%. Principal coordinate analysis (PCoA) plots were generated using Fast Unifrac. Enriched and depleted OTUs were identified using METASTATS [21]. The OTUs were obtained from Mothur, and were sorted from most to least abundant OTUs. Sequence abundance values within each OTU were normalized for comparisons of V3 OTU abundance between samples. Then, the sequence abundance values were log-transformed, and JMP was used for hierarchical clustering and visualization [22].

For metagenomic analysis, MG-RAST subsystem analysis was used to assign reads to functional groups using blastX and to identify bacterial taxa based upon metagenomic 16S rDNA reads [23]. MEGAN was used to assign total reads to taxonomic groups to all reads [24]. JMP was used for hierarchical clustering and visualization.

## Results

In total, 106,810 16S rRNA amplicon sequences were analyzed (Table 1). These reads were analyzed using two approaches: 1) classification of reads using the Ribosomal Database Project (RDP; http://rdp.cme.msu.edu/) [25]; and 2) assignment of reads to OTUs for analysis in the Mothur package [20].

**Table 1:** Number of OTUs per groups and estimators of sequence diversity and richness.

	# of Sequences	# of OTUs	Chao1 (richness)	Shannon (diversity)	Simpson (diversity:1-D)
Day 0 control^A^	4,872	259	430.7	3.2	0.91
Day 7 control	12,076	717	1267.9	4.3	0.96
Day 14 control	6,614	598	997.5	4.5	0.96
Day 35 control	7,023	678	1304.2	4.8	0.97
Day 0 monensin	3,006	105	216.4	2.2	0.73
Day 7 monensin	7,529	415	742.4	3.9	0.95
Day 14 monensin	12,987	783	1280.1	4.5	0.97
Day 35 monensin	1,944	348	556.5	4.9	0.99
Day 0 monensin + virginiamycin	7,504	379	620.6	3.7	0.94
Day 7 monensin + virginiamycin	8,797	538	877.8	4.3	0.96
Day 14 monensin + virginiamycin	7,882	690	1078.8	4.7	0.98
Day 35 monensin + virginiamycin	5,114	570	898.5	5	0.98
Day 0 monensin + tylosin	1,776	109	220.4	2.7	0.82
Day 7 monensin + tylosin	8,716	539	840.9	4.1	0.95
Day 14 monensin + tylosin	5,816	531	885.5	4.6	0.97
Day 35 monensin + tylosin	5,154	605	1056.5	5	0.98

A Day 0 samples were collected prior to the start of treatments. Subsequent days represent days post-treatment start.

### Taxonomic classification of 16S rRNA reads using RDP

**Figure 1:**
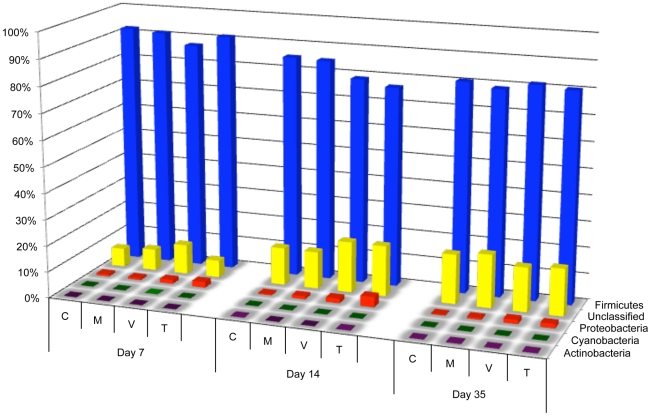
Bacterial phyla distributions at the three timepoints after the start of treatments, using V3 amplicon sequencing (n = 89,652). For each timepoint, C  =  control diet, M  =  monensin treatment, V  =  monensin/virginiamycin treatment, and T  =  monensin/tylosin treatment.

**Figure 2:**
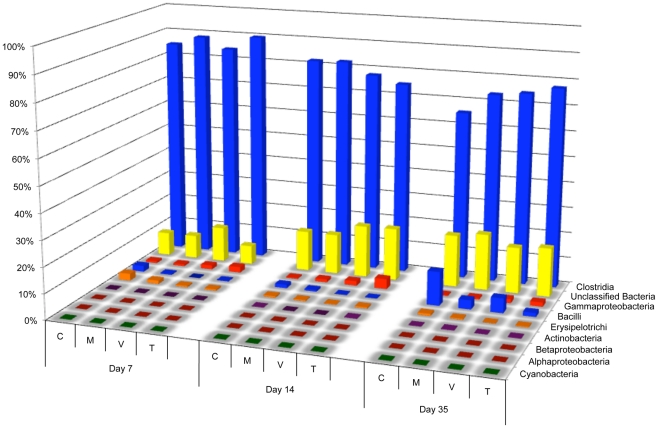
Bacterial class distributions among the three timepoints after the start of treatments, using V3 amplicon sequencing (n = 89,652). For each timepoint, C  =  control diet, M  =  monensin treatment, V  =  monensin/virginiamycin treatment, and T  =  monensin/tylosin treatment.

**Figure 3:**
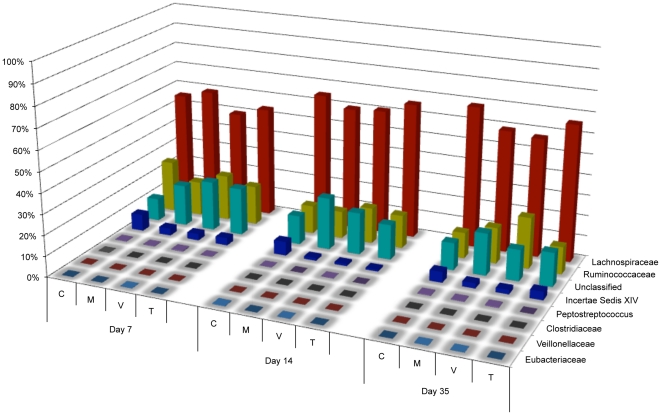
Bacterial family distribution within the *Firmicutes* phylum at the three timepoints after the start of treatment using V3 amplicon sequencing (n = 53,588). For each timepoint, C  =  control diet, M  =  monensin treatment, V  =  monensin/virginiamycin treatment, and T  =  monensin/tylosin treatment.

Sequence reads were analyzed on the phylum, class, order, family, and genus levels using the RDP database with a bootstrap confidence threshold of 80%. The dominant phylum at each timepoint was *Firmicutes*, comprising 75–90% of the samples throughout the experiment (Fig. 1 and Table S1). Using RDP's compare algorithm (Fig. S1), *Firmicutes* were found to be significantly depleted (p<0.05) in the day 7 and day 14 monensin/virginiamycin-treated groups and the day 14 monensin/tylosin-treated group, as compared to the control group for each respective timepoint. These reductions were not observed in the monensin-only treatment groups. Class distributions were also analyzed among the post-treatment timepoints (Fig. 2). The dominant class was *Clostridia*, followed by *Bacilli* and *Gammaproteobacteria*. In response to treatment, monensin alone acted to significantly reduce *Bacilli* at all three timepoints, an effect that was also observed for the monensin/virginiamycin and monensin/tylosin treatment groups. In contrast, monensin/virginiamycin and monensin/tylosin acted to increase *Gammaproteobacteria* at all three timepoints but this effect was not observed in the monensin-alone treatment groups (Fig. S1; p<0.05). Among the *Firmicutes*, the predominant families were *Lachnospiriceae*, *Ruminococcaceae*, and *Incertae Sedis XIV* (Fig. 3). A number of *Firmicutes* families were significantly decreased by monensin alone, and/or monensin/AGP treatment, including *Erysipelotrichaceae* at day 7 post-treatment in all groups, *Lactobacillaceae* by monensin alone at all timepoints, *Enterococcaceae* at day 14 by all groups, *Lachnospiraceae* at day 7 by all groups, and *Insertae Sedis XIV* by monensin alone at all timepoints (Fig. S1; p<0.05). On the genus level, there were a number of *Firmicutes* genera that were either significantly enriched or depleted by monensin and/or growth promoter treatment (p<0.05). *Roseburia* was significantly depleted at nearly all timepoints by all treatment types, compared to the control group. In contrast, *Escherichia* was significantly enriched at all timepoints in the virginiamycin- and tylosin-treated groups (Fig. S1).

### Comparison of 16S rRNA reads using OTU analysis

**Figure 4:**
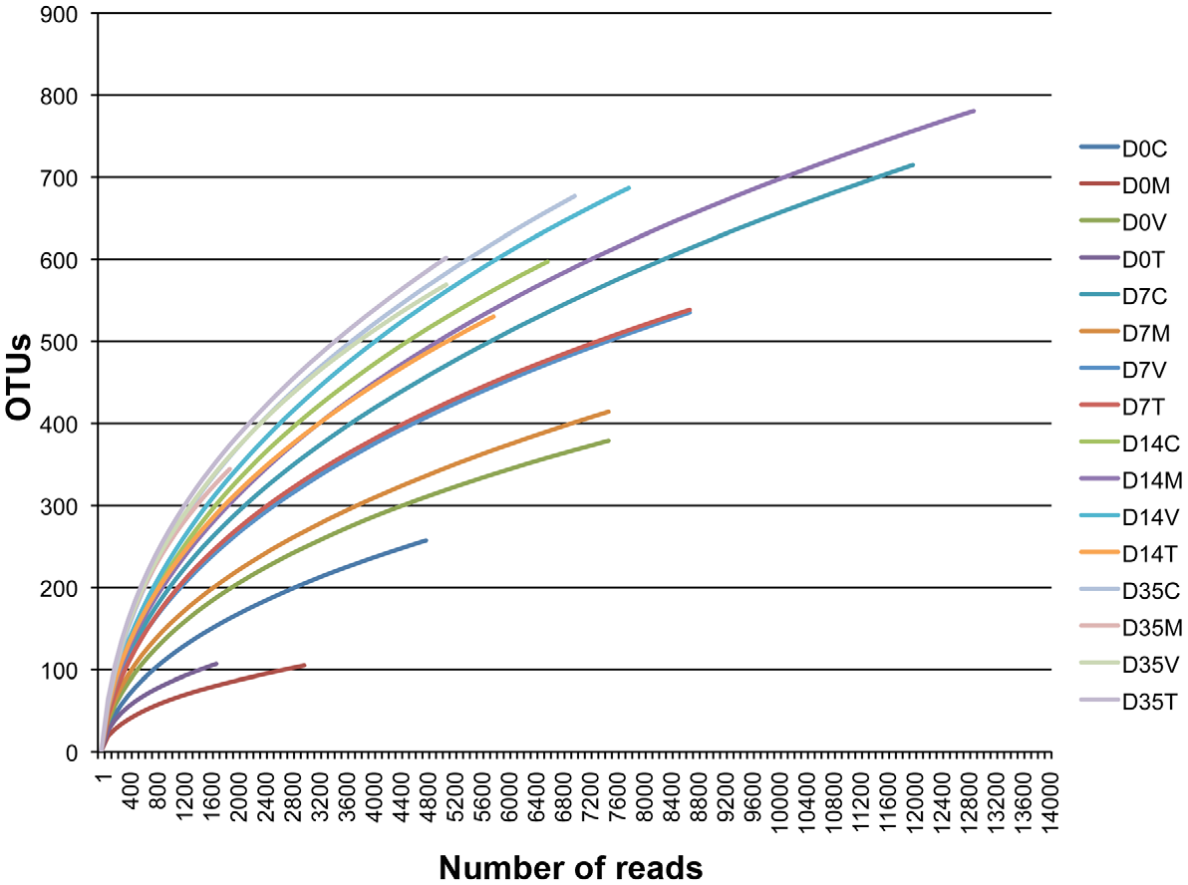
Rarefaction curves of samples from the different groups examined in this study using a cutoff value of 0.03. For each timepoint (D0, D7, D14, and D35), C  =  control diet, M  =  monensin treatment, V  =  monensin/virginiamycin treatment, and T  =  monensin/tylosin treatment.

**Figure 5:**
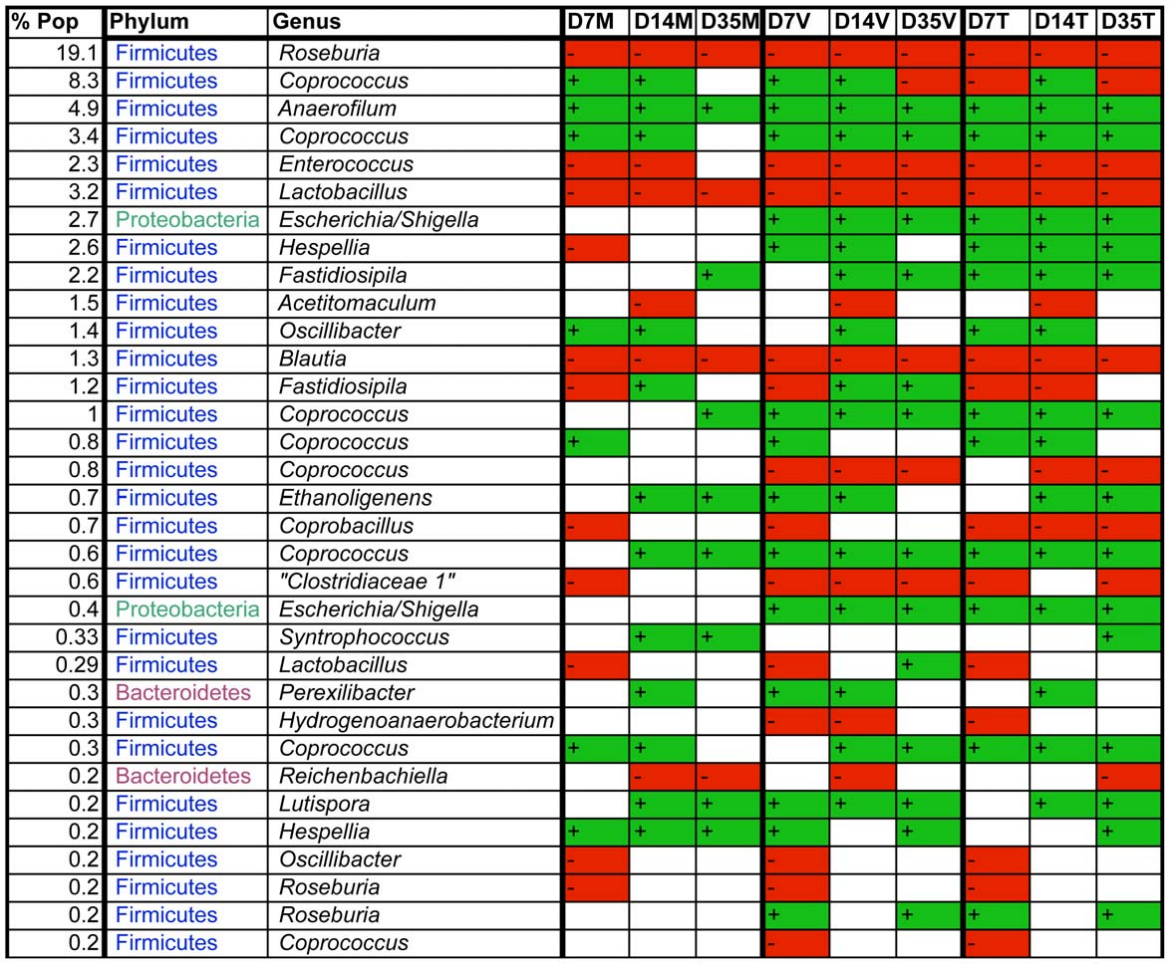
Most abundant OTUs identified in chicken cecum samples throughout all timepoints. Classifications of representative sequences from the OTU using RDP with their bootstrap confidence values are shown, as well as if an OTU was significantly enriched (green) or depleted (red) compared to the control group for that timepoint (p<0.05). For each timepoint (D7, D14, and D35), C  =  control diet, M  =  monensin treatment, V  =  monensin/virginiamycin treatment, and T  =  monensin/tylosin treatment.

The 16S rRNA sequence reads were also binned according to their sequence similarities with one another, and independent of any database hits or searches. With an OTU definition at a similarity cut-off of 95%, a total of 2,304 OTUs were identified among the 16 different groups examined. There was an overall increase in the number of OTUs identified per group as the bird aged (Table 1). This was also reflected by the Chao1, Shannon, and Simpson analyses of sample richness and diversity, which suggested that sample richness and diversity increased with the increasing age of the bird. Rarefaction analysis of the experimental groups agreed with this, as the slopes of the curves increased with increasing bird age (Fig. 4). Each of the 2,203 OTUs were analyzed for significant enrichments or depletions in treatment groups, as compared to the control groups of the same timepoint, then OTUs with significant changes (p<0.05) were sorted by abundance and classified using RDP (Fig. 5 and Table S2). A number of OTUs were uniformly affected across treatment groups and/or timepoints. OTUs that were significantly and uniformly depleted included those classified as *Roseburia*, *Enterococcus*, *Lactobacillus*, and *Blautia*. OTUs that were significantly and uniformly enriched included those classified as *Anaerofilum*, *Coprococcus*, *Lutispora*, and *Hespellia*. There were also OTUs that were only enriched or depleted in the virginiamycin/tylosin groups but not the monensin group relative to control groups within the same timepoints, such as those classified as *Fastidiosipila*, *Escherichia*, and *Hespellia*.

**Figure 6:**
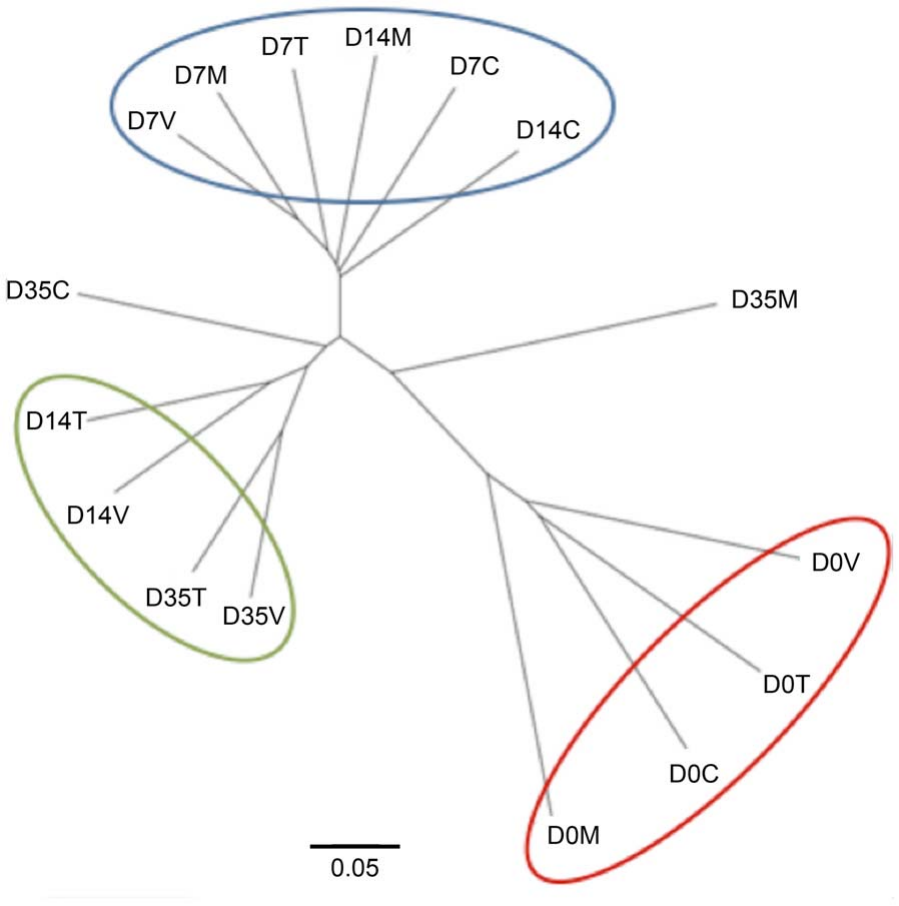
Dendrogram depicting relationships among the experimental groups in this study using OTU analysis, generated using Bray-Curtis index. Circled clusters represent arbitrary groupings showing the groups that are most similar to one another. For each timepoint (D0, D7, D14, and D35), C  =  control diet, M  =  monensin treatment, V  =  monensin/virginiamycin treatment, and T  =  monensin/tylosin treatment.

**Figure 7:**
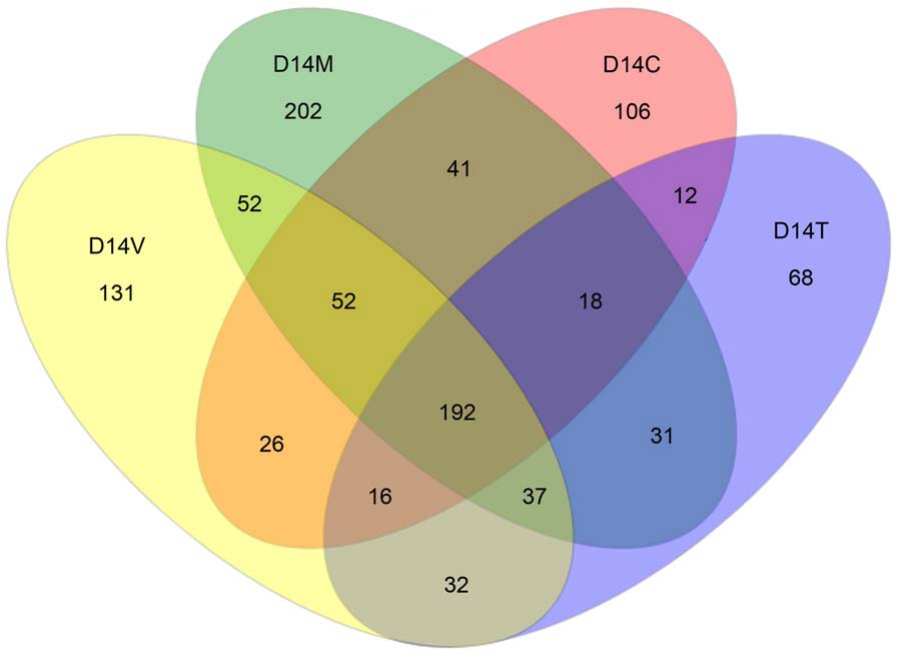
Venn diagram illustrating shared and unique OTUs at day 14 days after the start of treatments. Numbers below groups indicate the number of OTUs within each sector. For each timepoint, C  =  control diet, M  =  monensin treatment, V  =  monensin/virginiamycin treatment, and T  =  monensin/tylosin treatment.

The OTU composition across groups was further analyzed for similarities in community structure using the Bray-Curtis index. In the resulting dendrogram, groups tended to cluster by bird age (Fig. 6). However, the day 14 control and monensin-treated groups clustered with all day 7 groups, whereas the day 14 monensin/virginiamycin and monensin/tylosin treatment groups clustered with the day 35 groups. A PCoA plot was also generated using all of the amplicon sequencing reads, and the samples were predominantly clustered according to bird age although the treatment groups at later timepoints also clustered separate from control groups of the same timepoint (Fig. S2). Venn diagrams were constructed to depict shared and unique OTUs among the groups examined at each timepoint. At 14 days after the start of treatment, 192 (18.9%) OTUs were shared among all groups studied, while 507 (49.9%) were unique to one of the four different treatment groups (Fig. 7). RDP classification of the unique OTUs belonging to the monensin/virginiamycin and monensin/tylosin treatment groups revealed that most of the sequences were classified within the family *Ruminococcaceae*, including the genera *Anaerotruncus*, *Subdoligranulum*, and *Sedimentibacter*, all absent from the control group.

### Changes in cecum microbiome microbial community structure over time

**Figure 8:**
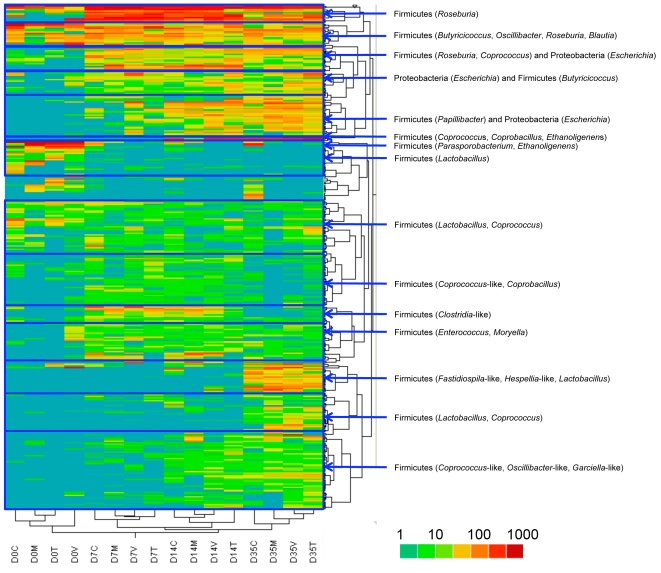
Hierarchical clustering of OTU similarity and group similarity using normalized abundances. Key genera are highlighted to the right of each cluster. For each timepoint (D0, D7, D14, and D35), C  =  control diet, M  =  monensin treatment, V  =  monensin/virginiamycin treatment, and T  =  monensin/tylosin treatment.

Two-way hierarchical clustering was also performed to depict the relationships between OTUs and experimental groups based upon sequence abundance within each OTU (Fig. 8). Here, changes in OTU structure based upon bird age were evident, with certain OTUs present in the cecum of the young birds that disappeared over time, OTUs that emerged in the cecum of birds of older ages, and some OTUs that were present throughout the production lifespan of the chicken. For instance, the most abundant OTUs with RDP classification as *Roseburia*, *Coprococcus*, *Butyricicoccus*, *Escherichia*, and *Papillibacter* appeared at 14 days of age (day 7 post-treatment) and persisted through 42 days of age (day 35 post-treatment). Some OTUs with RDP classification as *Lactobacillus*, *Parasporobacterium*, and *Ethanoligenens* were present prior to the start of treatments but disappeared at later timepoints. Some OTUs with RDP classification as *Firmicutes*, such as the genera *Butyricicoccus*, *Oscillibacter*, *Roseburia*, and *Blautia* were consistently present throughout all timepoints. Furthermore, as the chicken gut diversified, older birds of all groups acquired OTUs classified as genera *Fastidiospila*, *Hespellia*, *Lactobacillus*, and *Coprococcus*.

### Chicken cecum metagenome changes in response to growth promoter treatment

Shotgun metagenome sequencing was performed on samples at days 14 and 35 post-treatment to identify changes in the metabolic potential of the cecum microbial population in response to monensin, monensin/virginiamycin, and monensin/tylosin treatments (Table 2). A total of 1,291,219 reads with average lengths ranging from 234–399 bp were generated spanning the eight groups and timepoints sequenced. The proportion of bacterial sequences in this sample was estimated at 94–97% based upon reads from the metagenomic dataset, with the remainder of these reads belonging primarily to Archaea and Eukarya (Table 2).

**Table 2:** Summary of shotgun metagenome sequencing of chicken cecum samples.

	Day 14 control	Day 14 monensin	Day 14 monensin + tylosin	Day 14 monensin + virginiamycin	Day 35 control	Day 35 monensin	Day 35 monensin + tylosin	Day 35 monensin + virginiamycin
Number of reads	128,982	305,528	115,681	182,848	156,320	163,732	121,864	116,264
Total size (bp)	32,338,847	79,616,004	27,138,472	71,548,774	62,175,491	65,442,527	47,477,308	45,454,330
Average read length	250.72	260.58	234.6	391.3	397.7	399.7	389.6	390.9
Plasmids (%)	0	0	0.1	0.1	0	0	0.1	0
Eukaryota (%)	2.1	3.4	1.4	2.2	0.5	0.5	0.7	0.6
Bacteria (%)	95.6	94.2	96.1	95.4	97.1	97.2	96.8	96.9
Viruses (%)	0.2	0.2	0.4	0.3	0.3	0.3	0.3	0.3
Archaea (%)	2.1	2.1	1.9	2.1	2.1	2	2.1	2.2

**Figure 9:**
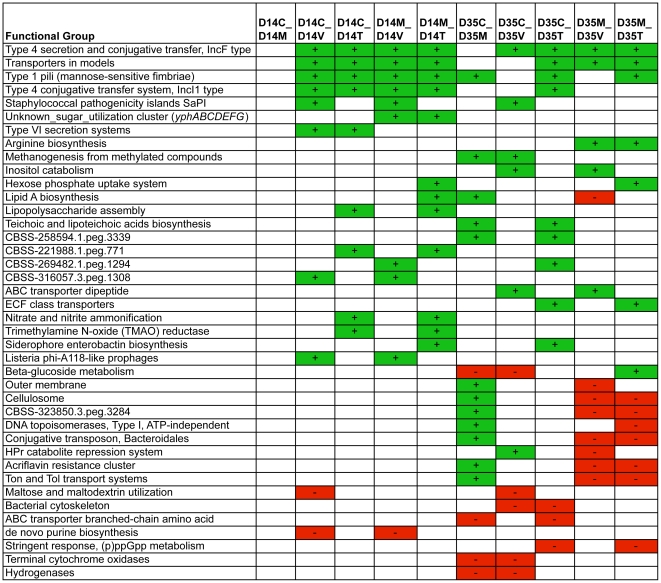
Comparison of functional group distribution identified via shotgun metagenomic sequencing at timepoints D14 and D35. Only groups with multiple significant shifts (p<0.05) are shown, from a total of 773 functional subsystems. Functional groups that were significantly enriched (green) or depleted (red) compared to the control group for that timepoint are shown (p<0.05). For each timepoint (D0, D7, D14, and D35), C  =  control diet, M  =  monensin treatment, V  =  monensin/virginiamycin treatment, and T  =  monensin/tylosin treatment.

MG-RAST was used to bin the sequences into functional groups on three different subsystem levels. Pairwise comparisons were then performed between control versus treatment groups of the same timepoint, and between monensin alone versus monensin/AGP treatment groups of the same timepoint. On the broadest level containing 29 different subsystems, no significant changes (p<0.05) were observed between any of the groups examined (Fig. S3). The most prevalent functional groups to which the sequences were binned included carbohydrate utilization, clustering-based subsystems (functional coupling evidence but unknown function), protein metabolism, and amino acid synthesis (Fig. S4). When analyzed on the most focused subsystem level containing 773 functional groups, a number of significant changes (p<0.05) were observed between the control versus monensin treatment groups, and between the monensin versus monensin/AGP treatment groups. The most significant changes observed in the control and/or monensin versus monensin/AGP treatment comparisons included sequence enrichments in subsystems containing ‘transporters in models’, type IV secretion systems, and type I pili (Fig. 9 and Fig. S5). The ‘transporter in models’ group included reads with similarity to a variety of bacterial species, with predicted proteins such as amino acid carrier proteins, iron transport system proteins, potassium/sodium efflux proteins, magnesium transport system proteins, uncharacterized ABC-type transporter systems, heavy metal and antimicrobial transport system proteins, and sugar transport system proteins. The type IV secretion system subgroup included genes from IncF and IncI1 plasmids with predicted protein hits to the conjugative transfer systems of these plasmids. The type I pili group included reads mostly with similarity to *E. coli*, and included predicted proteins matching their type I fimbrial components. Analysis of all functional classes of antimicrobial resistance genes revealed no significant differences between the control and treatment groups in the binned sequences within each subsystem class (Table 3).

**Table 3:** Summary of resistance-associated subsystems among shotgun metagenomic reads.

Class	D14C^A^	D14M	D14V	D14T	D35C	D35M	D35V	D35T
Multidrug resistance efflux pumps	1948	1972	2042	1817	2103	1955	2141	1995
Fluoroquinolone resistance	545	496	435	516	425	459	377	395
Cobalt-zinc-cadmium resistance	287	358	427	271	391	427	382	362
Tetracycline resistance, ribosome protection	224	267	269	261	253	242	256	250
Beta-lactam resistance	120	148	131	127	119	176	99	155
Vancomycin resistance	113	121	107	106	114	107	122	117
Vancomycin tolerance in *Streptococcus pneumoniae*	59	52	71	20	117	73	104	94
Acriflavin resistance	47	53	61	43	58	138	48	61
Streptothricin resistance	36	37	45	38	24	17	25	27
Integrons	27	22	36	28	29	46	33	29
Methicillin resistance in *Staphylococcus*	27	22	19	20	27	26	10	19
Multidrug resistance, tripartite systems in Gram-negative bacteria	20	16	30	28	8	33	20	27
Arsenic resistance	16	9	19	18	15	19	10	5
Multidrug resistance, 2-protein systems in Gram-positive bacteria	16	24	30	8	42	22	15	24
Multidrug resistance (MdtABCD)	16	11	20	20	6	5	12	14
Colicin E2 tolerance	14	15	31	10	10	17	23	21
USS-DB-2	11	5	15	8	4	5	10	8
Aminoglycoside resistance	7	8	20	10	7	5	5	5
Multiple antibiotic resistance Mar locus	7	5	12	5	6	6	8	8
Zinc resistance	7	21	12	25	18	45	30	24
Multidrug efflux pump in *Campylobacter jejuni* (CmeABC)	5	1	12	0	6	4	2	3
USS-DB-1	5	13	5	15	12	14	18	10
Mercuric reductase	2	2	3	5	1	2	7	2
Mercury resistance	0	0	1	5	0	0	2	2
MexA-MexB-OprM multidrug efflux	0	0	0	0	0	0	3	0
Teicoplanin resistance in *Staphylococcus*	0	0	1	3	1	0	0	3
USS-DB-6	0	1	0	0	0	0	2	0

Raw counts were normalized to day 14 monensin group total read counts.

A D14C  =  Day 14 control; D14M  =  Day 14 monensin; D14V  =  day 14 monensin + virginiamycin; D14T  =  D14 monensin + tylosin; D35C  =  Day 35 control; D35M  =  Day 35 monensin; D35V  =  day 35 monensin + virginiamycin; D35T  =  35 monensin + tylosin.

## Discussion

With increasing pressures to reduce or eliminate the use of antimicrobials in production animals, there is a growing need to better understand the effects elicited by these agents in order to identify alternative approaches that might be used to maintain animal health. Antibiotic usage at subtherapeutic levels is postulated to result in modulations to the microbes within the gut, resulting in the suppression of bacterial pathogens, reduction of nutrient use by the microflora, increased production of vitamins and other nutrients by the microflora, and reduced production of ammonia by the microflora [26]. Here, we studied the effects of a monensin/AGP regimen typical of that applied to broilers.

Treatment with monensin alone acted to affect a number of bacterial genera within the chicken cecum. Monensin acted to significantly deplete sequences classified as *Roseburia*, an effect that was also observed in the monensin/AGP treatment groups. One of the depleted OTUs classified as *Roseburia* was by far the most abundant OTU identified, representing 19.1% of all binned sequences. *Roseburia* is known as a butyrate-producing organism, belonging to the *Lachnospiraceae* family, with a high capacity to form conjugated linoleic acid from linoleic acid [28], [29], [30]. Conjugated linoleic acid has been shown to exhibit anti-obesetic and anti-diabetogenic properties [28]. Recently, *Roseburia* was shown to be negatively correlated with mouse obesity; that is, *Roseburia* spp. were restored in the cecal contents of mice treated to revert from an obese to non-obese state [28]. In other studies, this genus has also been identified as a key player in dietary changes related to an obese versus non-obese state [31], [32] and has been negatively correlated with growth performance in production pigs [33]. While this evidence is circumstantial, it is possible that a reduction in *Roseburia* could promote weight gain in birds or more simply serve as an indicator of growth-promoting effects on the gut microbial level.

In contrast to the *Roseburia* depletions, monensin and monensin/AGP treatment significantly enriched five OTUs at most timepoints that were classified as *Coprococcus*, which is also a butyrate-producing member of the *Lachnospiraceae* family [34]. The exact reasons for a depletion of *Roseburia* and a corresponding increase in *Coprococcus* are unclear, but could represent the occupation of an available niche within the gut resulting in an overall balance of *Firmicutes* belonging to the *Lachnospiraceae* family. Another abundant OTU identified as enriched by monensin and monensin/AGP treatments was classified as the genus *Anaerofilum*. *Anaerofilum* is a genus of the *Ruminococcaceae* family containing strictly anaerobic, gram-positive bacteria [35] but is poorly described in the literature. Therefore, it is difficult to gauge the possible impact that the enrichment of this OTU might have in the chicken cecum microbial community.

Monensin treatment alone acted to significantly deplete the most abundant *Lactobacillus* OTU, representing 3.2% of the total binned sequences. Previous work has demonstrated that the use of growth promoters and additive dietary enzymes act to reduce lactobacilli populations in the ileum and cecum [36], [37] as does the use of monensin [38]. In addition to the *Lactobacillus* OTUs, an abundant OTU classified as *Enterococcus* was also depleted in response to monensin, monensin/virginiamycin, and monensin/tylosin treatments. In a controlled experiment such as that performed here, this might be expected because enterococci can be susceptible to ionophores, virginiamycin, and tylosin. However, in poultry production environment utilizing growth promoters, multidrug resistant enterococci are common [39], [40], so these results might not be extended to the use of AGPs in production settings. It has been shown, though, that *Enterococcus* resistance to virginiamycin is not affected by the use of subtherapeutic levels of the drug in feed, so it is unclear if these levels would actually drive the persistence of resistant enterococci clones [41], [42].

While the experimental design used in this study prevented us from determining the precise effects of growth promoters alone, some changes in the microbiome were observed in the monensin/AGP treatment groups that were not seen in the monensin-alone treatment group. The most apparent of these changes were significant enrichments in sequences classified as *E. coli*. Previous culture-dependent studies have not observed an effect on *E. coli* populations in response to growth promoters [43]. However, the growth promoters used should not have a spectrum of activity that includes *E. coli*, so this was not necessarily a surprising finding. Some other abundant OTUs were identified as unique to the monensin/virginiamycin and monensin/tylosin treatment groups as compared to the monensin and control groups at day 14 post-treatment start. These included the genera *Anaerotruncus* and *Subdoligranulum*, which are gram-positive, anaerobic non-spore-forming bacteria [44], [45], and *Sedimentibacter*, which is a spore-forming, gram-positive anaerobe [46]. *Subdoligranulum* spp. have been shown to be enriched under fructo-oligosaccharide treatment in piglets [47] and have been associated with “healthy-specific” bacterial sequences identified in humans in a study of Crohn's disease [48]. The implications of these unique microbes in growth-promoting microbial health are unclear, however they could potentially be used as markers of a healthy gut state. Overall, a number of bacterial taxa were modulated through the use of monensin/virginiamycin and monensin/tylosin, but the cause and effect relationships driving these shifts remain to be determined.

At all timepoints and treatment groups, *Firmicutes* was the predominant phylum identified within the chicken cecum, similar to what has been previously described [49], [50]. Few studies have previously examined the longitudinal succession of microbes in the chicken GI tract. A study by Lu et al. examined the succession of microbes in the ileum and cecum of chickens fed diets devoid of any coccidiostat or growth promoting agent [49]. They found that *Firmicutes* dominated the chicken cecum throughout the grow-out phase of the bird, and a large proportion of the *Firmicutes* they identified belonged to the *Clostridium* genus (29–46%) with few or no identified *Proteobacteria*. The chickens assessed in our study generally lacked *Clostridium*, and were instead predominated by sequences belonging to the *Lachnospiraceae*, *Ruminococcaceae*, and *Incertae Sedis IV* families. Many factors could contribute to these differences, including different diets, bird type, environment, and rearing, as well as differences in technical methodologies. These complexities make it difficult and unjustified to compare with other studies in this manner. Evident from this work, though, is that bird age and gut maturation had a much greater effect on microbiome than did treatment effects. We observed an increase in the complexity of the chicken cecum microbiome over time, with a shift from apparently transient to stable populations, similar to previous work [49]. The clustering approach further clarified the diversification of the chicken cecum of the aging bird, with more OTUs emerging over time than those disappearing. The cecum microbiome at days 14, 21, and 42 of age were considerably more complex than day 7 birds (Table 1 and Fig. 8), underscoring the finding that the chicken cecum is simplified but transient in the young bird. Also, it was evident that changes occurred with respect to bacterial clones classified within the same genus, with some OTUs replacing or supplementing others classified within the same genus. Overall, OTU analysis showed that the effects of monensin/AGP treatment were subtle compared to gut maturation effects. However, the greatest effects of our monensin/AGP treatments were observed at 14 days after the start of treatment, and monensin/virginiamycin and monensin/tylosin treatment appeared to modulate the cecal microbiome towards a more mature state with microbiome compositions more closely resembling later age timepoints.

It has previously been suggested that the “core” microbiome in the gut is not dictated by the actual bacterial species present, but by the collective functional traits that this community contains [49]. The results of this study support the concept that “what they are doing,” not “who is there,” likely best defines the core gut microbiome. We observed no differences in the chicken cecum communities in response to age or anticoccidial and growth promoter treatments when analyzed on the broadest functional classifications. However, significant differences were detected using the most focused subsystem classifications. The functional groups that were identified as significantly enriched in the metagenomes of monensin/virginiamycin and monensin/tylosin treatment groups, but not in the monensin-treated groups, included IncF and IncI1 type IV conjugative secretion systems, type I fimbrial systems, and transporter systems. IncI1 and IncF plasmid types are most common among *E. coli*; thus the increase in gene sequences encoding these type IV secretion systems is likely an effect of *E. coli* enrichment [51], [52]. Similarly, the enrichment of type I fimbrial sequences is likely attributed to the increase in *E. coli* populations. In contrast, the ‘transporters in models’ subsystem that was significantly enriched by monensin/virginiamycin and monensin/tylosin treatment contained sequences with BLAST similarity across many taxa. The predicted proteins of this group had various biological processes, including the transport of amino acids, iron and manganese, potassium and sodium, sugars, heavy metals, and calcium. Modification of the availability of transport systems in a microbial community might improve the range of carbon sources available for utilization, increase metabolic precursor availability for the synthesis of amino acids and metabolic intermediates, increase the efficiency in sugar mixture utilization through catabolic repression, and control overflow metabolism resulting in reduced acetate production [53].

The spectrums of activity of virginiamycin and tylosin are somewhat similar. Virginiamycin is a streptogramin with a narrow spectrum of activity that includes gram-positive bacteria (i.e., staphylococci, streptococci, and enterococci) and some gram-negative cocci [26]. Genes associated with virginiamycin resistance include *vat*(*A–E*), *vgb*(*A*), *vga*(*A*), and *mrs*(*A*) [26]. Tylosin is a macrolide-class antibiotic with broad-spectrum activity against gram-positive bacteria and a limited spectrum of activity against gram-negative bacteria. Genes associated with macrolide resistance include the *erm* genes encoding ribosomal methylases, the *mef* and *msr* genes encoding for efflux proteins [27]. We searched our metagenomic datsets for these virginiamycin and tylosin resistance genes, and they were present in all treatments and timepoints examined but did not differ significantly in their distribution between groups. Furthermore, analysis of the shotgun metagenomic dataset for all antibiotic resistance-associated subsystems detected no significant differences in the distribution of these subsystems among the control versus treatment groups, suggesting that subtherapeutic treatment with virginiamycin and tylosin did not enrich for resistance-associated genes in these short-term controlled experiments (Table 3). This finding may not be extendable to the commercial poultry environment, though, since different sources of microbes and differences in selective pressures in these environments could contribute to the emergence of drug resistant microorganisms.

Overall, this study identified a number of significant modulations within the chicken cecum in response to monensin alone, monensin/virginiamycin, and monensin/tylosin treatment. Some of these identified changes might help to explain why the use of growth promoters and anticoccidials results in improved health and weight gain. However, these identified changes are descriptive in nature, therefore it is unclear if the modulated bacteria are playing a role in the benefits conferred through gut microbial modulation, if they are artifacts, or if they are markers of a modulated gut that confers health benefits to the host. A limitation to this study is that it was performed only in the chicken cecum. Future work should also include locations in the upper GI tract to determine the modulations that occur there, since they are also likely important in the overall health of the avian GI tract. Also, this study was performed in a controlled animal facility experiment, thus the microbes encountered in this environment are likely much different than those encountered in poultry production. Finally, samples from multiple animals and experiments were pooled, negating the ability to assess animal-to-animal and experiment-to-experiment variation. However, recent high throughput sequencing of commercial pigs revealed that the fecal microbiota of individual pigs within the same farm converges over time, suggesting that animal-to-animal variation could be minimal in genetically similar production animals, and that environment plays a larger role in determining the fate of the production animal gut microbiome [54]. Nevertheless, this study provides a more comprehensive glimpse at the gut microbial modulations in response to growth promoters in poultry, and provides future targets and markers with which to mimic the effects of growth promoters using alternative approaches.

## Supporting Information

Figure S1 **Analysis of bacterial taxa within each group and timepoint using the RDP Database.** For each timepoint, taxa that are significantly enriched (green) or depleted (red) compared to control groups are depicted (p<0.05). For each timepoint, C  =  control diet, M  =  monensin treatment, V  =  monensin/virginiamycin treatment, and T  =  monensin/tylosin treatment.
(TIF)

Figure S2 **PCoA plot of similarities between the different timepoints and treatments examined.** For each timepoint, C  =  control diet, M  =  monensin treatment, V  =  monensin/virginiamycin treatment, and T  =  monensin/tylosin treatment.
(TIF)

Figure S3 **Distribution of functional groups from shotgun metagenome sequencing, using the broadest functional subsystem classification in MG-RAST (n = 29).** For each timepoint, C  =  control diet, M  =  monensin treatment, V  =  monensin/virginiamycin treatment, and T  =  monensin/tylosin treatment.
(TIF)

Figure S4 **Breakdown of functional group distributions at D14C using subsystem analysis in MG-RAST.** All other timepoints and treatments were similar in their distributions.
(TIF)

Figure S5 **Two-way hierarchical clustering of 773 functional group subsystems identified using MG-RAST, based upon normalized abundances.** For each timepoint (Day 14 and Day 35), C  =  control diet, M  =  monensin treatment, V  =  monensin/virginiamycin treatment, and T  =  monensin/tylosin treatment.
(TIF)

Table S1 **Bacterial phyla distributions (%) at the three timepoints after the start of treatments.** For each timepoint, C  =  control diet, M  =  monensin treatment, V  =  monensin/virginiamycin treatment, and T  =  monensin/tylosin treatment.
(XLSX)

Table S2 **Most abundant OTUs with significant changes in response to anticoccidial/growth promoter treatment.** See Fig. 5 for description of table.
(XLSX)
