# Supplementary figures and images for: Modulations of the Chicken Cecal Microbiome and Metagenome in Response to Anticoccidial and Growth Promoter Treatment
Source: PLoS One. 2011 Nov 16;6(11):e27949. doi: 10.1371/journal.pone.0027949 (PMC3218064; doi:10.1371/journal.pone.0027949)

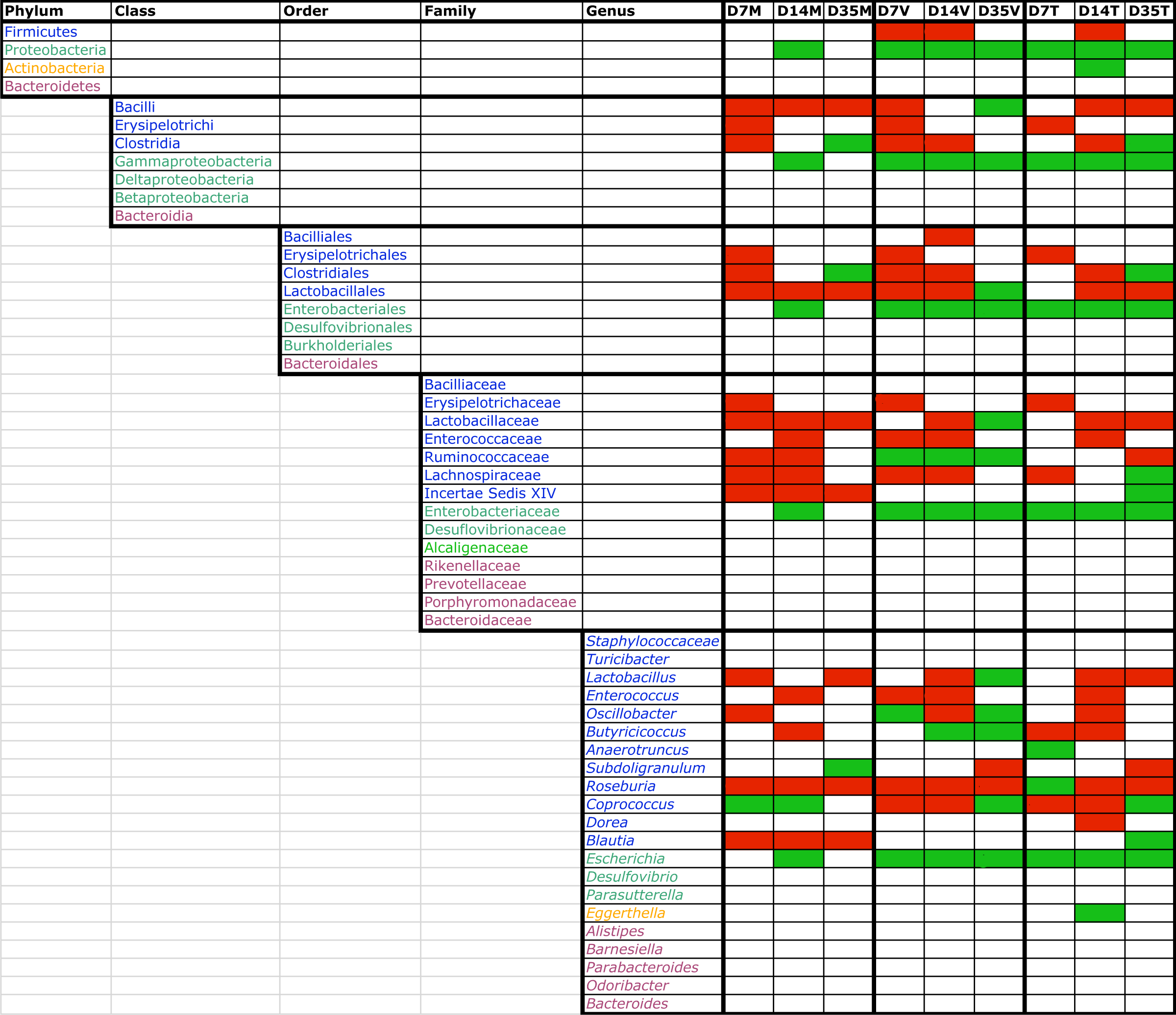

Supplement: Figure S1 — Analysis of bacterial taxa within each group and timepoint using the RDP Database. For each timepoint, taxa that are significantly enriched (green) or depleted (red) compared to control groups are depicted (p<0.05). For each timepoint, C = control diet, M = monensin treatment, V = monensin/virginiamycin treatment, and T = monensin/tylosin treatment. (TIF) [file pone.0027949.s001.tif]

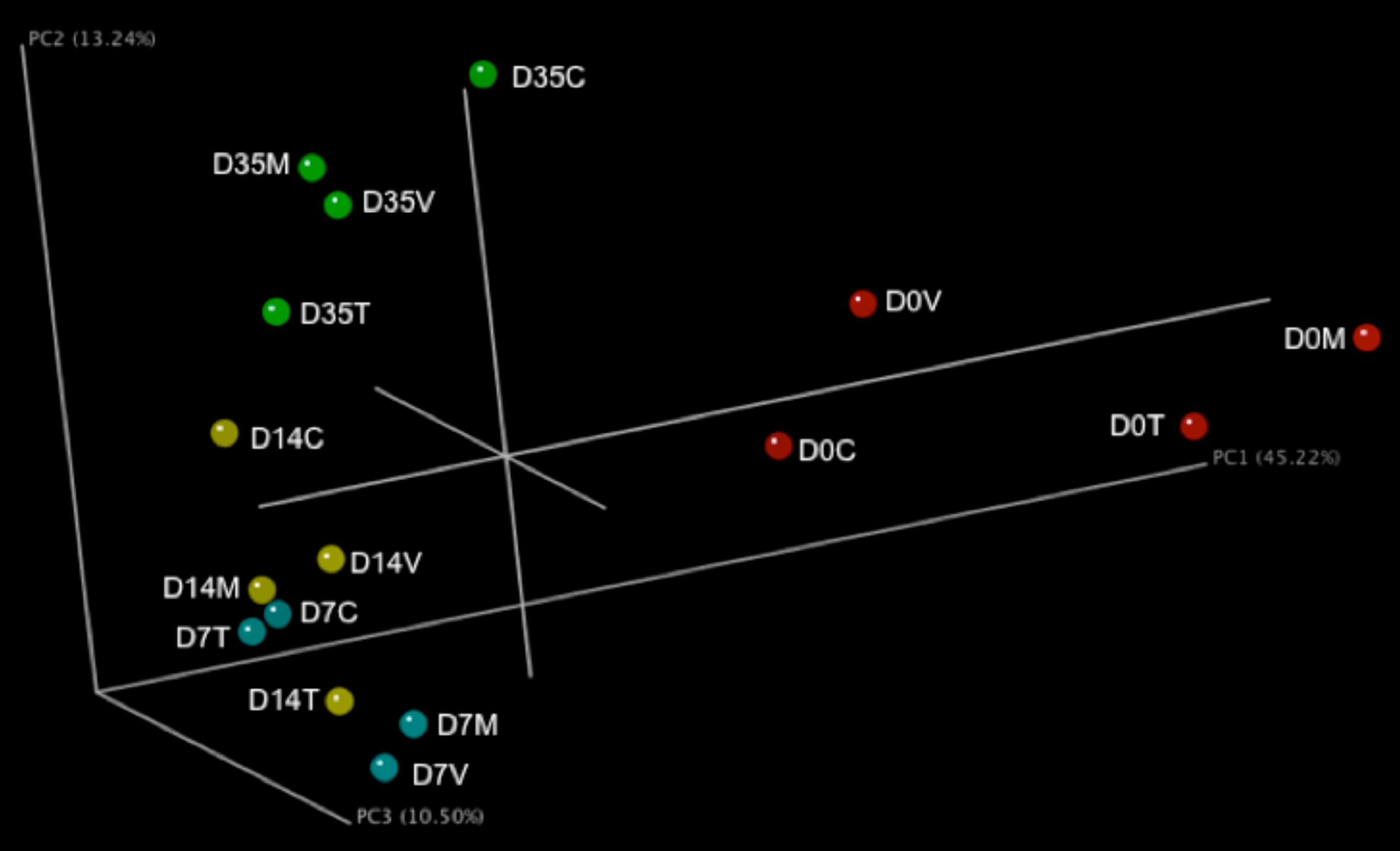

Supplement: Figure S2 — PCoA plot of similarities between the different timepoints and treatments examined. For each timepoint, C = control diet, M = monensin treatment, V = monensin/virginiamycin treatment, and T = monensin/tylosin treatment. (TIF) [file pone.0027949.s002.tif]

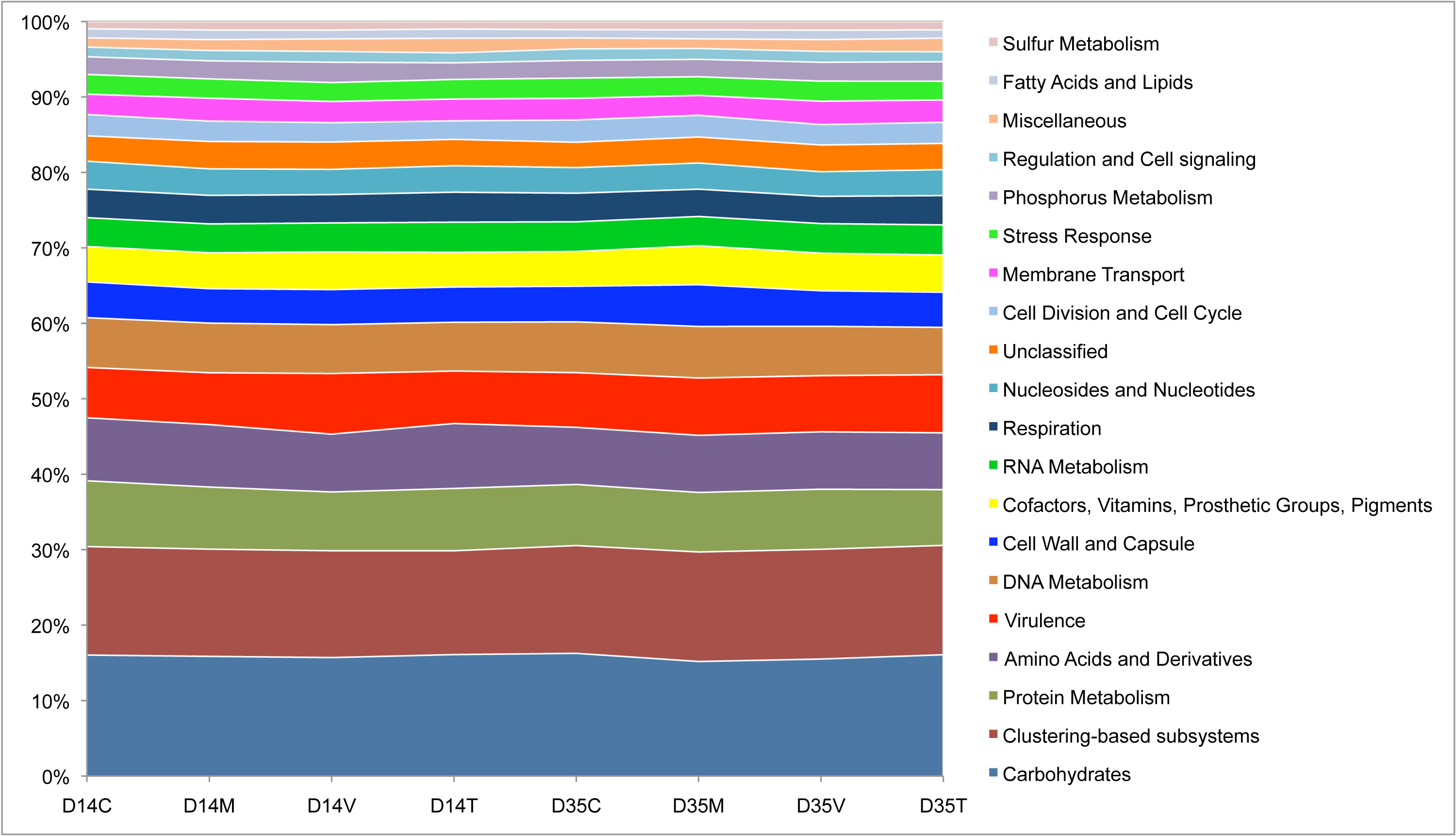

Supplement: Figure S3 — Distribution of functional groups from shotgun metagenome sequencing, using the broadest functional subsystem classification in MG-RAST (n = 29). For each timepoint, C = control diet, M = monensin treatment, V = monensin/virginiamycin treatment, and T = monensin/tylosin treatment. (TIF) [file pone.0027949.s003.tif]

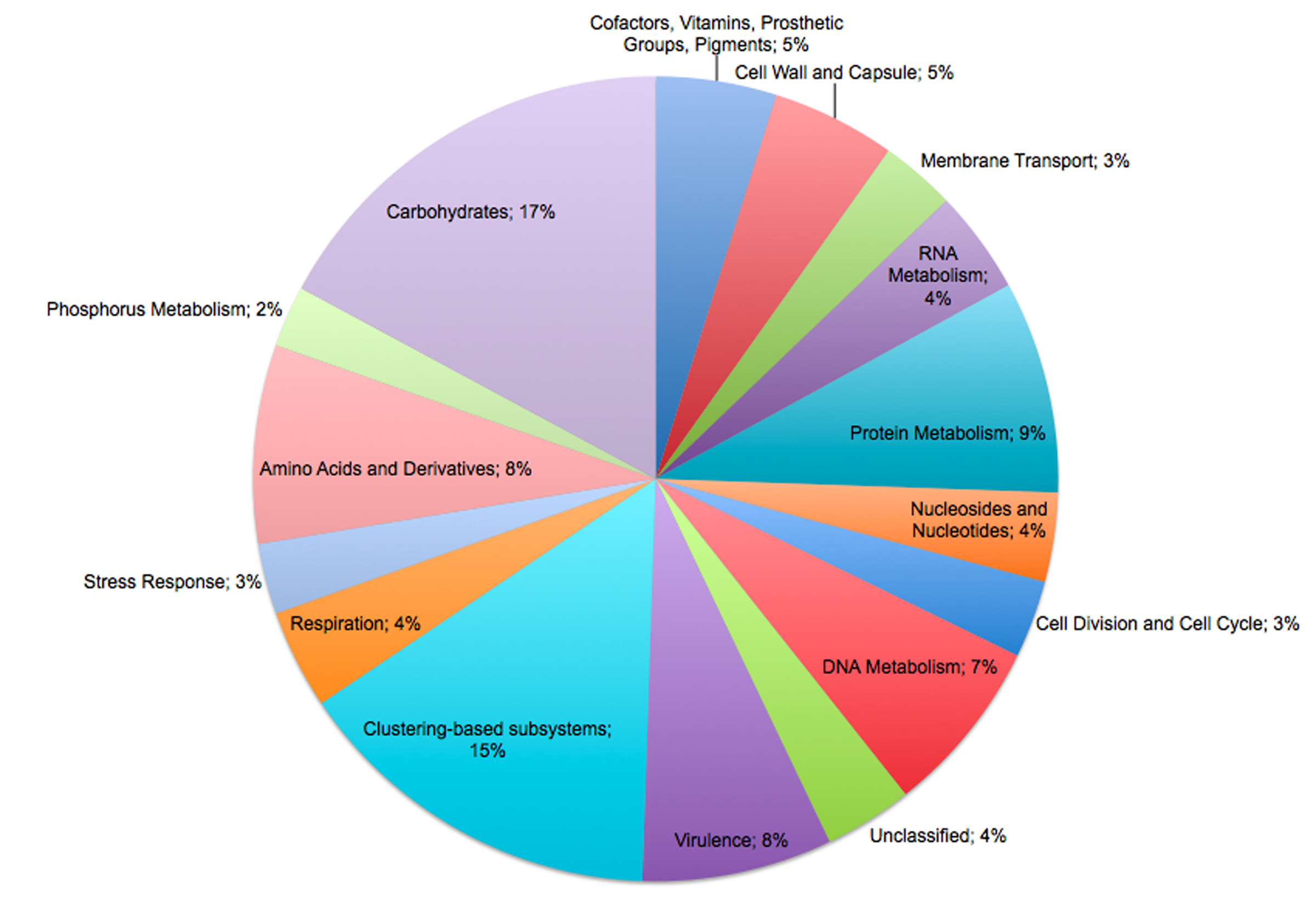

Supplement: Figure S4 — Breakdown of functional group distributions at D14C using subsystem analysis in MG-RAST. All other timepoints and treatments were similar in their distributions. (TIF) [file pone.0027949.s004.tif]

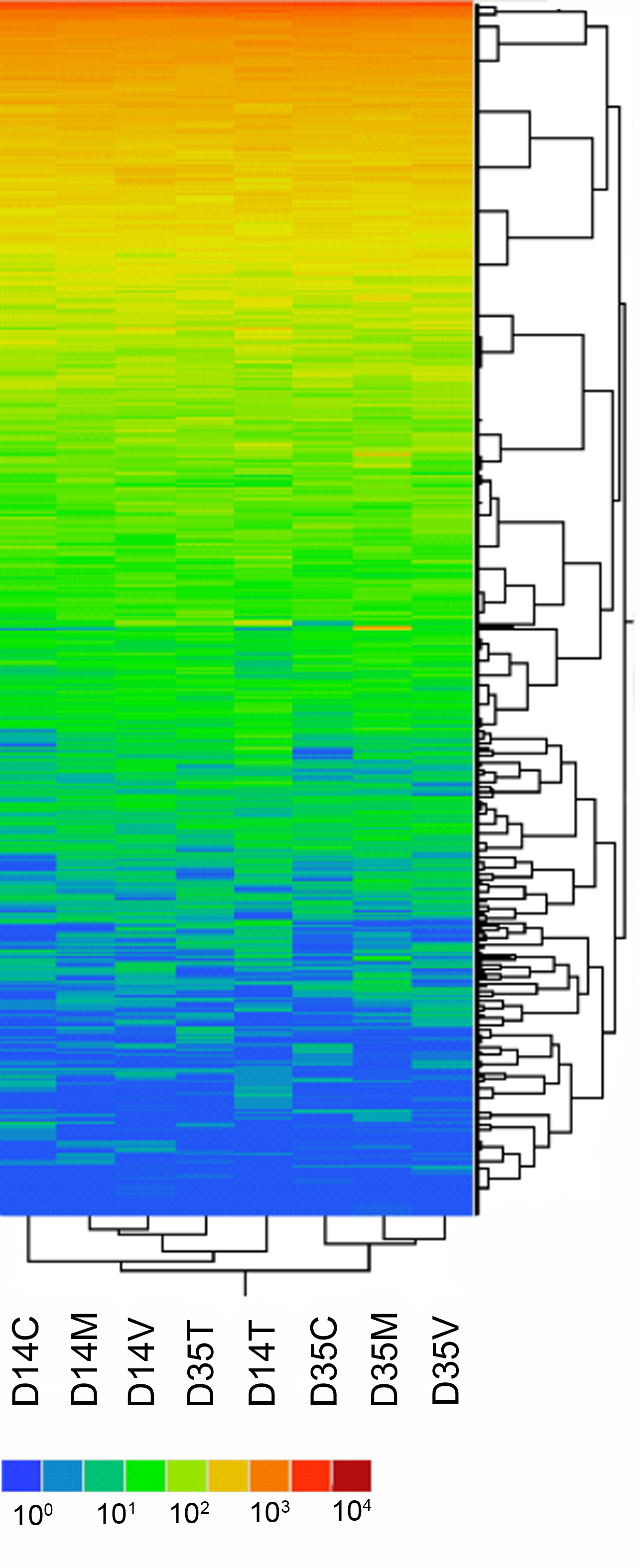

Supplement: Figure S5 — Two-way hierarchical clustering of 773 functional group subsystems identified using MG-RAST, based upon normalized abundances. For each timepoint (Day 14 and Day 35), C = control diet, M = monensin treatment, V = monensin/virginiamycin treatment, and T = monensin/tylosin treatment. (TIF) [file pone.0027949.s005.tif]
